# Best practices in methodological frameworks for evaluating procedure-based treatments in traditional, complementary and integrative medicine: a scoping review protocol

**DOI:** 10.1136/bmjopen-2025-110327

**Published:** 2025-12-24

**Authors:** Yaping Liu, Shuo Cui, Yushan Zhang, Ziwei Song, Zhenshan Luo, Zhongjie Chen, Qi Gao, Jingjing Wang

**Affiliations:** 1 Institute of Acupuncture and Moxibustion, China Academy of Chinese Medical Sciences, Beijing, Beijing, China中国中医科学院针灸研究所，北京，中国，北京

**Keywords:** COMPLEMENTARY MEDICINE, Acupuncture, Methods

## Abstract

**Abstract:**

**Introduction:**

While numerous evidence-based studies have been conducted on procedure-based treatments (PBTs) in traditional, complementary and integrative medicine (TCIM) (eg, acupuncture, special diets, lifestyle modification, *yoga, Tai χ*), high-quality research reports accepted by the academic community remain scarce. Key factors contributing to the low evidence quality in this field include researchers’ insufficient grasp of clinical research methodology concepts, inadequate study designs and lack of pilot studies. Scholars now widely recognise that establishing a robust evaluation framework for PBTs in TCIM is crucial for progressively refining research protocols and advancing clinical practice. Therefore, this scoping review aims to systematically map current evaluation methods for PBTs in TCIM, analyse their critical procedural components and lay the groundwork for developing a tailored evaluation framework.

**Methods and analysis:**

Forty-three databases will be systematically searched using comprehensive search strategies. Two independent reviewers will screen potential literature and select eligible studies. Literature management will be performed using NoteExpress and Excel 2016, with a pre-designed standardised Excel sheet employed for data extraction.

This scoping review will include literature that provides multidimensional evaluation (eg, efficacy, safety and health economics) for PBTs in TCIM. This encompasses methodological guidelines, systematic protocols outlining evaluative structures, procedural steps and core components, as well as conceptual or theoretical frameworks describing phased evaluation processes. Screening and data extraction will be conducted independently by two researchers. Inter-rater agreement will be assessed using the Kappa statistic. Any discrepancies will be resolved through consultation with a senior reviewer or correspondence with original authors.

Data extraction will capture: general information, types and number of included primary studies, interventions assessed, evaluation dimensions, procedural workflows for evaluation, version iterations of evaluation frameworks, staging configurations for evaluation, framework development methods, as well as documented strengths and limitations of the frameworks.

Results will be structured following the Preferred Reporting Items for Systematic reviews and Meta-Analyses extension for Scoping Reviews checklist. Findings will be presented through tables, charts and figures, with narrative synthesis describing key outcomes.

**Ethics and dissemination:**

No private information was issued in the entire process of the systematic review. Therefore, ethical approval is not required. Findings of the scoping review will be published in a peer-reviewed journal and/or disseminated through conference presentations.

**PROSPERO registration number:**

The protocol has been archived in the Open Science Framework (Registration DOI: https://doi.org/10.17605/OSF.IO/92DRM).

**STRENGTHS AND LIMITATIONS OF THIS STUDY**

- Comprehensive search across 43 databases and multiple languages enhances coverage, reducing geographic/language bias.
- Use of established scoping review methodologies (Joanna Briggs Institute and Preferred Reporting Items for Systematic reviews and Meta-Analyses extension for Scoping Reviews) ensures rigour.
- Inclusion of grey literature and official guidelines improves contextual relevance.
- Limitation: non-English literature may be under-represented despite translation efforts.
- Limitation: heterogeneity in framework reporting may challenge synthesis.

## Introduction

 Procedure-based treatments (PBTs) in traditional, complementary and integrative medicine (TCIM) refer to a therapeutic approach wherein a traditional medicine doctor prescribes an individualised, often multidimensional, programme according to a patient’s needs. Such interventions may include combinations of herbal medicine(s), acupuncture, special diet, lifestyle modifications, *yoga, tai χ* or *qigong*, and Panchakarma with distinct formulations for each patient.^1^

Evaluation of PBTs in TCIM constitutes a focal point and critically debated issue in traditional medicine development. Despite substantial evidence-based research in this field,^2^,^4^ high-quality studies accepted by the academic community remain limited.^5^,^7^ Scholars attribute this gap to several factors,^8^,^10^ including insufficient researcher command of clinical methodology concepts,^11^,^14^ suboptimal study designs,^15 16^ contradictions between research outcomes in high-impact publications and clinical realities^17^ and absence of preliminary studies and premature initiation of randomised controlled trials (representing additional contributors to diminished evidence quality.^18^ Contemporary scholars widely recognise that establishing a scientifically rigorous evaluation framework for PBTs in TCIM is paramount for progressively refining research protocols and advancing clinical applications.

Scoping reviews represent systematic evidence-mapping methodologies that identify key concepts,^19^ evidence distribution patterns and research gaps through comprehensive literature synthesis, thereby providing foundational insights for policy development and further investigation. At the request of and under supervision by the WHO, this scoping review will encompass multidimensional evaluations of PBTs in TCIM, including efficacy, safety and health economics.

Therefore, this scoping review aims to systematically map current evaluation frameworks for PBTs in TCIM, analyse their critical procedural components and establish a methodological foundation for developing a tailored evaluation framework aligned with the unique characteristics of PBTs in TCIM.

### Objectives

The primary objective of this scoping review is to systematically map the global evidence landscape for multidimensional evaluation (eg, efficacy, safety, health economics) of PBTs in TCIM across WHO Member States. Specific objectives include:

1. To identify the dimensional metrics currently employed in the multidimensional evaluation (eg, efficacy, safety, health economic) of PBTs in TCIM.
2. To analyse procedural steps, staging configurations and stage-specific objectives within existing evaluation frameworks for PBTs in TCIM.
3. To compare and contrast methodological commonalities and variations in the evaluation of different PBTs in TCIM (eg, qigong vs acupuncture vs yoga) across dimensions.
4. To examine how current methodologies integrate traditional empirical knowledge with modern scientific standards across all assessment dimensions.

## Methods

The proposed scoping review will be conducted following the Joanna Briggs Institute (JBI) methodology for scoping reviews.^20^ The methodological framework will adhere to the five-stage process developed by Arksey and O’Malley,^21^ which consists of: (1) Identifying the research question, (2) Identifying relevant studies, (3) Selecting studies, (4) Charting the data and (5) Collating, summarising and reporting the results. The review process will be reported in accordance with the Preferred Reporting Items for Systematic reviews and Meta-Analyses extension for Scoping Reviews (PRISMA-ScR) checklist.

And guided by the JBI,^22^ this protocol has been registered in the Open Science Framework (https://osf.io/92drm).^23^ The study is scheduled to run from August 2025 to April 2026, detailing a structured timeline as follows: protocol development and finalisation in August 2025; literature retrieval in September 2025; literature screening from October to December 2025; data extraction in January–February 2026; data analysis in March 2026 and completion of all study activities by April 2026.

### Stage 1: identifying the research question

This scoping review aims to systematically identify, map and analyse existing methodological frameworks used for the evaluation of PBTs in TCIM.

The primary research question guiding this review is: what are the existing methodological frameworks and their component evaluation methods (including those addressing efficacy, safety and health economics) for assessing PBTs in TCIM?

Aligned with this primary question, the following secondary questions will further guide the inquiry:

1. What are the specific metrics and instruments currently used to evaluate PBTs across the dimensions of efficacy, safety and health economics?
2. What are the key structural and procedural components of these evaluation frameworks, including their recommended stages, workflows and version iterations?
3. How do these frameworks integrate traditional empirical knowledge with contemporary scientific standards in their evaluation approaches?

The purpose of this review is to comprehensively summarise and analyse the scope, structure and application of these evaluation frameworks. This will help identify methodological gaps and commonalities and provide a robust evidence base to inform the future development of a standardised, multidimensional evaluation framework tailored to the unique complexities of PBTs in TCIM.

### Stage 2: identifying relevant studies

#### Inclusion and exclusion criteria

The inclusion and exclusion criteria for this scoping review will be presented following the four key dimensions of Participants, Concept, Context and Types of Sources,^24^ with detailed criteria presented in table 1.

**Table 1:** Study participants, concept, context and types of evidence

	Inclusion	Exclusion
Participants	Evidence from articles aimed at providing evaluation for PBTs in traditional, complementary and integrative medicine, regardless of disease type	Literature irrelevant to PBTs in TCIM
Concept	This review will focus on evaluation methodological frameworks explicitly designed for the multidimensional assessment (efficacy, safety and health economics) of PBTs in TCIM. This includes methodological guidelines, systematic protocols and conceptual/theoretical frameworks that define evaluative structures, procedural steps or core components	Literature that does not explicitly describe or propose an evaluation framework/method
Context	This review will examine literature from global sources without geographical restrictions, covering the period from database inception to 1 August 2025. Frameworks from international organisations (eg, WHO, INAHTA) will be prioritised. The context is strictly limited to literature pertaining to PBTs within the domain of TCIM	No
Types of sources	Methodological guidelines: definitive documents from authoritative bodies/expert panels specifying phased evaluation procedures (eg, WHO guidelines, NICE handbooks, CONSORT extensions). Frameworks: systematic schemas delineating evaluative structures, procedural steps and core components (eg, UK MRC framework for complex interventions). Consensus statements and equivalent methodological resources	Literature without full-text access. Specific applications of frameworks/guidelines/models (eg, clinical studies, literature reviews using them). Original research: RCTs, non-RCTs, case series, etc. Animal studies. Publications in languages other than English (only English version retained). Publications available in multiple language versions (only English version retained)

CONSORT, Consolidated Standards of Reporting Trials; INAHTA, International Network of Agencies for Health Technology Assessment; NICE, National Institute for Health and Clinical Excellence; PBTs, Procedure-based treatments; RCTs, randomised controlled trials; TCIM, traditional, complementary and integrative medicine.

#### Search strategy

The search strategy will aim to locate both published and unpublished studies. A three-step search strategy will be used in this review.

Firstly, an initial search of PubMed, MEDLINE, Scopus and China National Knowledge Infrastructure (CNKI) will be conducted to determine if any relevant articles or documents exist.

Secondly, based on the text words contained in the titles and abstracts, as well as the keywords or index terms of the articles obtained from the initial search, this review develops a list of keywords and conducts a comprehensive search across forty-three electronic databases, including seven English databases, five Chinese databases, one Russian database, one French database, four German databases, two Spanish databases, two Japanese databases, five Korean databases, one Arabic database and fifteen Evidence-based medicine and guideline databases. The databases to be systematically searched will include Pubmed, Medline, EMBASE, Web of Science, Scopus, NCCIH, Cinahl, CNKI, Wanfang Data, SinoMed, VIP China Science and Technology Journal Database, Scientific Information Database, Elibrary.ru, BibCNRs, EBSCO, De Gruyter, DNB, DigiZeitschriften, Dialnet, FECYT, JSTAGE, CINII, KoreaMed, ScienceON, National Assembly Library of the Republic of Korea, Research Information Sharing Service, Korean Journal Database, Almanhar, DynaMed, Trip Database, Cochrane, PROSPERO, Evidence-Based, Medicine Reviews, Bandolier, Health Technology Assessment, NHS, Health Systems Evidence, Database of Abstracts of Reviews of Effects, National Guideline Clearinghouse, Guidelines International Network, National Institute for Health and Clinical Excellence, Scottish Intercollegiate Guidelines Network.

Thirdly, supplemental searches of the reference lists of selected literature will be performed. Comprehensive reports issued by official agencies will also be searched, such as academic degree dissertations from universities, conference papers from academic conferences and systematic review registration protocols from the PROSPERO website; additionally, the following websites will also be searched to identify relevant unpublished sources including:

- WHO guidelines (https://www.who.int/).
- World Federation of Acupuncture-Moxibustion Societies (http://wfas.org.cn/).
- Google Scholar (https://scholar.google.cz/).
- Canada’s Drug Agency (https://www.cda-amc.ca/).
- The independent Institute for Quality and Efficiency in healthcare (https://www.iqwig.de/en/).
- The International Network of Agencies for Health Technology Assessment (https://www.inahta.org/).
- ICMART (https://icmart.org/).
- ProQuest Dissertations and Theses Global (https://about.proquest.com/en/products-services/pqdtglobal/).

Materials will be searched from inception to 1 August 2025. A combination of MeSH terms and free-text keywords will be used for the search. The search terms include: acupuncture, moxibustion, traditional Chinese medicine, complementary and complementary medicine, traditional medicine, procedure-based treatment, massage, chiropractic, osteopathy, manual therapies, qigong, tai ji, yoga, naturopathy, thermal medicine, physical therapies, mental therapies, spiritual therapies, mind-body therapies, framework, guidance, roadmap, model, tool, method, evaluate. An example of the search strategy is provided in table 2.

**Table 2:** Search strategies of PubMed

Search	Search fields	Query
#1	Title/Abstract	traditional medicines, psychosomatic medicine, naturopathy, non-pharmacological therapies, acupuncture, acupuncture therapy, electroacupuncture, moxibustion, cupping therapy, auriculotherapy, ear acupuncture, auricular plaster therapy, pricking blood therapy, acupressure, bloodletting, filiform needle, three-edged needle, TEAS, transcutaneous electrical acupoint stimulation, scalp acupuncture, plum-blossom needle, seven-star needle, catgut embedding, dermal needle, fire needle, moxa cone, acupotomy, scraping therapy, *Tai χ, Yijinjing, Gua Sha*, *Qigong*, *Baduanjin, Liuzijue,* meditation, breathing therapy, yoga, chiropractic, osteopathy, manipulation, energy therapy, music therapy, reiki, dance therapy, thermal medicine, complex interventions, multimodal therapies
#2	Title/Abstract	framework, guidance, roadmap, model, tool, method, evaluate, assessment, appraisal
#3	Title/Abstract	animals, rats, mice, experiment, animal experiment
#4	Title/Abstract	#1 AND #2 NOT #3

The search strategy will be adjusted to suit each database. This search list may be refined considering the literature nature of the review process. Any changes in the searching process will be documented and reported in the results paper.

There will be no language restrictions in the literature selection process. To ensure conceptual and terminological accuracy of non-English sources, a rigorous multi-stage translation and validation protocol will be implemented, beginning with an initial machine-translated draft, followed by independent verification by two bilingual researchers with expertise in traditional medicine who will cross-check domain-specific terminology and conceptual nuance. Any disagreements between the validators will be resolved through consultation with a third senior researcher or, where feasible, by contacting the original authors for clarification.

### Stage 3: selecting studies

All identified citations will be collected and uploaded into NoteExpress software and duplicates will be removed. After an initial screening of the titles and abstracts, the citation information of the articles included will be exported to Excel 2016 for full-text screening based on the pre-specified inclusion and exclusion criteria in the review protocol.

Two reviewers (YL and SC) will independently perform the literature screening at all stages. To ensure consistent application of the selection criteria, a calibration exercise will be conducted prior to formal screening using a random sample of 50 citations. Both reviewers will independently evaluate the titles and abstracts of these articles based on the inclusion and exclusion criteria, classifying each as ‘yes’, ‘no’ or ‘uncertain’.

Inter-rater agreement will be measured using Cohen’s Kappa coefficient. A Kappa value of ≥0.6 is considered to indicate substantial agreement and will be deemed acceptable.^25^ If the initial Kappa value falls below this threshold, the reviewers will discuss discrepant items to clarify their interpretations and improve consensus. Should persistent ambiguities in the criteria be identified, the inclusion and exclusion criteria will be refined under the guidance of the project leader (JW). A repeat calibration test with a new sample may be performed to verify improved agreement.

Following the calibration process, the formal screening will proceed through two sequential phases. Initially, both reviewers will independently screen the titles and abstracts of all retrieved citations against the inclusion and exclusion criteria. Articles classified as ‘yes’ or ‘uncertain’ in this stage will proceed to full-text screening, where reviewers will independently assess their eligibility based on complete text examination. Any disagreements between reviewers at either screening stage will be resolved through discussion, with unresolved cases being adjudicated by the project leader (JW).

**Figure 1:**
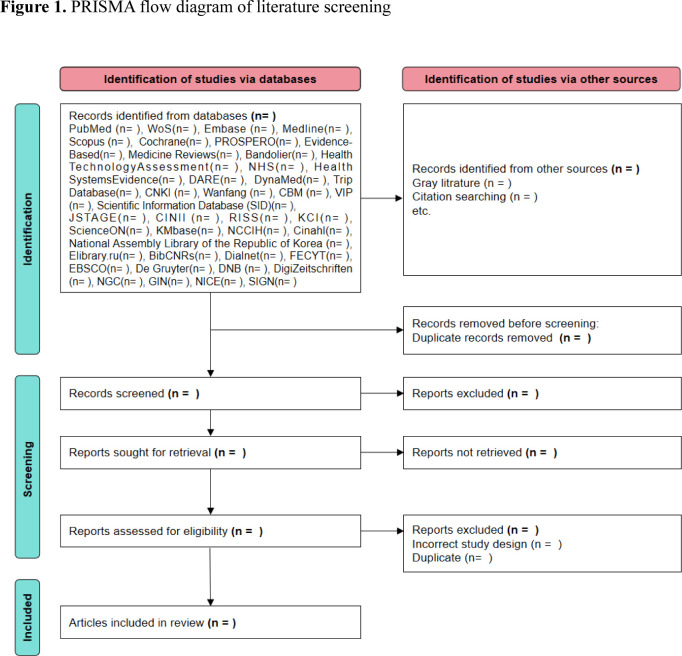
Preferred Reporting Items for Systematic Reviews and Meta-Analyses flow diagram of literature screening.

The results of the search and the study inclusion process will be reported in the final scoping review and presented in a PRISMA flow diagram.^26^ Figure 1 shows the PRISMA flow diagram of the process of literature screening and selection.

### Stage 4: charting the data

After literature search, the research team created an extraction form to extract data related to the RQs. A standardised Excel spreadsheet will be designed to load the information extracted from eligible literature. 50 eligible articles will be screened by two reviewers (YL and SC) to test the extraction form and ensure the consistency. If needed, the extraction form will be revised to capture sufficient details, and any modifications to the extraction table will be recorded in the final scoping review. When two reviewers have completed half of the extraction work, they will cross-check each other’s work. Any discrepancies will be discussed again. If necessary, a third review will assist in reaching a consensus. The data extraction form is presented in table 3.

**Table 3:** Extraction domains and examples

Classification	Subdivision	Examples
General information	Journal, author, country, language, year of publication, settings, etc	
Characteristics	Interventions	Acupuncture, moxibustion, traditional Chinese medicine, complementary and complementary medicine, traditional medicine, procedure-based treatment, massage, chiropractic, osteopathy, manual therapies, *qigong, tai ji, yoga*, naturopathy, thermal medicine, physical therapies, mental therapies, spiritual therapies, mind-body therapies, etc
	Framework iterations	For example, MRC framework, IDEAL framework
	Evaluation stage	Theory Modelling Exploratory trial Definitive randomised controlled trial Long-term implementation
	Framework development approach	Literature review, mechanistic research, expert consensus, adaptation of existing frameworks, etc
	Evaluation dimensions	Efficacy, safety, health economic, etc
	Strengths	For example, standardised procedures, comprehensive scope, broad applicability, enhanced reproducibility
	Limitations	For example, restricted adaptability, inadequate reflection of TCIM characteristics and constrained contextual relevance

TCIM, traditional, complementary and integrative medicine.

### Stage 5: collating, summarising and reporting the results

During this stage, extracted data will be synthesised using a mixed-methods approach to address both descriptive and analytical aims of the scoping review. The process comprises the following components:

#### Quantitative analysis: profiling framework characteristics

Data will be systematically organised and analysed using descriptive statistics to characterise the landscape of existing frameworks. This analysis will quantify key categorical variables including publication year, geographical distribution, TCIM traditions and coverage of evaluation dimensions (efficacy, safety, health economics). Relationships between framework attributes—such as development approaches and evaluation stages—will be examined through cross-tabulation analysis. Results will be presented through summary tables, statistical charts and accompanying narrative descriptions to provide a comprehensive evidence profile.

#### Qualitative analysis: examining structural and comparative features

Thematic analysis will be employed to systematically identify and categorise framework components, procedural elements and structural configurations. This process will examine how different frameworks integrate traditional empirical knowledge with modern scientific standards, while conducting comparative assessment of their strengths, limitations and applicability for PBT evaluation in TCIM contexts. Through iterative data examination, the analysis will identify critical features contributing to effective evaluation approaches, with particular emphasis on elements that successfully bridge traditional practices and contemporary research methodologies.

#### Reporting the results

Findings will be reported following PRISMA-ScR guidelines^27^ and will include:

- A PRISMA flow diagram detailing document identification, screening and inclusion/exclusion processes.
- A consolidated summary of existing iterations of evaluation methods for PBTs in TCIM.
- A descriptive analysis of staging configurations within evaluation methodologies for PBTs in TCIM.
- An inductive synthesis of structural commonalities in staging frameworks across diverse evaluation approaches.
- A comparative assessment of methodological strengths and limitations identified in the included frameworks

### Ethics and dissemination

No private information was issued in the entire process of the systematic review. Therefore, ethical approval is not required. For confirmation, the Scientific Research and Education Office of the Institute of Acupuncture and Moxibustion, China Academy of Chinese Medical Sciences, which oversees research affairs, can be contacted via kychzhen@163.com.

Findings of the scoping review will be published in a peer-reviewed journal and/or disseminated through conference presentations.
