# Supplementary material for: Best practices in methodological frameworks for evaluating procedure-based treatments in traditional, complementary and integrative medicine: a scoping review protocol
Source: BMJ Open. 2025 Dec 24;15(12):e110327. doi: 10.1136/bmjopen-2025-110327 (PMC12742101; doi:10.1136/bmjopen-2025-110327)
Supplement: Supplementary file 1 [file bmjopen-2025-110327.reviewer_comments.pdf]

PEER REVIEW HISTORY

BMJ Open publishes all reviews undertaken for accepted manuscripts. Reviewers are asked to complete a checklist review form and are provided with free text boxes to elaborate on their assessment. These free text comments are reproduced below.

ARTICLE DETAILS

Title (Provisional)

Best practices in methodological frameworks for evaluating procedure-based treatments in traditional, complementary, and integrative medicine: A Scoping Review Protocol

Authors

Liu, Yaping; Cui, Shuo; Zhang , Yushan; Song , Ziwei; Luo , Zhenshan; Chen, Zhongjie; Gao, Qi; Wang, Jingjing

---

VERSION 1 - REVIEW

**Reviewer**	**1**
**Name**	**najibi, seyede maryam**
**Affiliation**	**Shiraz University of Medical Sciences, Department of Persian Medicine, School of Medicine**
**Date**	**14-Oct-2025**
**COI**	**None**

---

this scoping review protocol presents a well-structured and ambitious plan to map methodological frameworks for evaluating Procedure-Based Treatments (PBTs) in Traditional, Complementary, and Integrative Medicine (TCIM). The reviewers collectively acknowledge the project's high potential for impact, given the clearly articulated research gap, the comprehensive, multilingual search strategy, and the significant endorsement from the World Health Organization (WHO). Adherence to established guidelines like PRISMA-ScR and JBI provides a strong foundation for methodological rigor. However, several critical areas require attention to elevate the protocol from good to excellent.

to translate 'any potential sources not in English... using Google Translate and cross-verified by two bilingual researchers' has significant methodological rigor and validity issues for capturing nuanced meanings in specialized methodological literature, for TCIM, where cultural and linguistic context is often deeply embedded in the methodology, relying solely on Google Translate risks misinterpreting conceptual novelty and the accuracy of insights from non-English sources, thus undermining the review's own claim to comprehensive and rigorous synthesis.

The protocol mentions a pilot screening of 50 articles to test for consensus, but it lacks a specific, quantifiable measure of agreement (e.g., Kappa statistic) and a clear action plan if consensus is not met. This ambiguity reduces the methodological transparency and rigor of the screening process.

Under 'Characteristics evaluation dimensions' in Table 3, the examples for 'Efficacy' ('pain Scores, functional improvement, changes in Inflammatory markers') primarily list specific outcome measures from primary studies rather than focusing on how methodological frameworks “propose to measure or address” these dimensions. This approach deviates from the review's core objective of mapping evaluation “frameworks” and risks, extracting tangential information, thereby diminishing the analytical rigor by misdirecting the focus during data charting.

The inclusion of 'Evaluation Tools, e.g., PRISMA' in the extraction domains is problematic because these are tools for assessing and reporting “primary studies”, not for evaluating the methodological rigor or quality of “methodological frameworks” themselves. The protocol lacks explicit criteria or a defined approach for critically appraising the rigor and quality of the identified frameworks, despite the stated objective of finding 'best practices'. This omission compromises the analytical validity and utility of the review, as it provides no mechanism to distinguish robust frameworks from less rigorous ones, thereby failing to fully achieve its 'best practices' aim, a critical gap in methodological transparency

---

VERSION 1 - AUTHOR RESPONSE

Reviewer Comments (Dr. seyede maryam najibi):

Comment 1: ...to translate 'any potential sources not in English... using Google Translate and cross-verified by two bilingual researchers' has significant methodological rigor and validity issues... **Response:** We thank Dr. seyede maryam najibi for highlighting this critical methodological issue. In response, we have completely revised the translation protocol. It now incorporates a rigorous multi-stage process, beginning with machine translation, followed by independent verification by bilingual TCIM experts, and an adjudication mechanism for resolving discrepancies (Pages 7).

Comment 2: The protocol mentions a pilot screening of 50 articles... but it lacks a specific, quantifiable measure of agreement (e.g., Kappa statistic)...

Response: We appreciate the reviewer’s valuable input on enhancing methodological transparency. We have now added a formal consistency check using Cohen’s Kappa coefficient during the pilot screening phase, along with a clear protocol for resolving disagreements through discussion and consultation with a senior researcher (Pages 7 to 8).

Comment 3: Under 'Characteristics evaluation dimensions' in Table 3, the examples for 'Efficacy'... primarily list specific outcome measures from primary studies rather than focusing on how methodological frameworks "propose to measure or address" these dimensions.

Response: We sincerely thank Dr. seyede maryam najibi for this critical insight. Following this suggestion, we conducted pilot screening which confirmed that the original extraction setup failed to capture key methodological features. We have therefore comprehensively revised Table 3 to better align with our research objectives. The updated form now enables extraction of fundamental framework characteristics through these newly added domains: 'evaluation stage,' 'framework development approach,' 'evaluation dimensions,' 'strengths,' and 'limitations.' These modifications allow us to more accurately capture how frameworks

conceptually approach evaluation dimensions rather than recording specific outcome measures (Pages 8 to 9).

Comment 4: The protocol lacks explicit criteria or a defined approach for critically appraising the rigor and quality of the identified frameworks...

Response: We fully acknowledge the importance of evaluating framework quality. However, after conducting a comprehensive literature review, we confirmed that there are currently no universally accepted, validated tools specifically designed for assessing the quality of methodological frameworks themselves—a recognized methodological gap in the field. In the absence of such standardized instruments, we have implemented a systematic qualitative assessment approach. This involves the comparative analysis of key framework attributes—including development methods, structural features, and documented strengths and limitations—to allow for a rigorous and practical evaluation of their relative robustness and applicability within the current methodological landscape (Page 9 to 10).

We sincerely thank the editor and reviewers for their insightful comments, which have provided important guidance for our research and substantially improved the quality of this manuscript, and has significantly strengthened the methodological rigor and overall value of our work.
